# Infant feeding method and special educational need in 191,745 Scottish schoolchildren: A national, population cohort study

**DOI:** 10.23889/ijpds.v8i2.2257

**Published:** 2023-09-14

**Authors:** Lisa Adams, Jill Pell, Daniel Mackay, David Clark, Albert King, Michael Fleming

**Affiliations:** 1 University of Glasgow, Glasgow, United Kingdom; 2 Public Health Scotland, Edinburgh, United Kingdom; 3 Scottish Government, Edinburgh, United Kingdom

## Background

Infant breastfeeding has been associated with reduced incidence of childhood physical and mental health problems. This study investigated relationships between infant feeding methods and the risk of Special Educational Need (SEN).

## Methods

A retrospective population cohort of schoolchildren in Scotland was constructed by linking together health (maternity, birth, and health visitor records) and education (annual school pupil census) databases. Inclusion was restricted to singleton children, born in Scotland from 2004 onwards with available breastfeeding data and who attended local authority mainstream or special schools between 2009 and 2013. Generalised estimating equation models with a binomial distribution and logit link function investigated associations between infant feeding method at 6–8 weeks and any-cause and cause-specific SEN, adjusting for sociodemographic and maternity factors.

## Findings

Of 191 745 children meeting inclusion criteria, 126 907 (66·2%) were formula-fed, 48 473 (25·3%) exclusively breastfed, and 16 365 (8·5%) mixed fed. Overall, 23 141 (12·1%) children required SEN. Compared with formula feeding, mixed feeding and exclusive breastfeeding were associated with decreased any-cause SEN (OR 0·90, 95% CI 0·84–0·95; and 0·78, 0·75–0·82), and SEN attributed to learning disabilities (0·75, 0·65–0·87; and 0·66, 0·59–0·74), and learning difficulties (0·85, 0·77–0·94; and 0·75, 0·70–0·81). Compared with formula feeding, exclusively breastfed children had less communication problems (0·81, 0·74–0·88), social-emotional-behavioural difficulties (0·77, 0·70–0·84), sensory impairments (0·79, 0·65–0·95), physical motor disabilities (0·78, 0·66–0·91), and physical health conditions (0·74, 0·63–0·87). Feeding method was not significantly associated with mental health conditions or autism.

## Interpretation

Many women struggle to exclusively breastfeed for the full 6 months recommended by WHO; however, this study provides evidence that a shorter duration of nonexclusive breastfeeding could nonetheless be beneficial with regard to the development of SEN.

